# Day-case stapes surgery: Day-case versus inpatient stapes surgery for otosclerosis: a randomized controlled trial

**DOI:** 10.1186/s12901-016-0024-6

**Published:** 2016-02-27

**Authors:** Laura S. M. Derks, Inge Wegner, Rinze A. Tange, Digna M. A. Kamalski, Wilko Grolman

**Affiliations:** Department of Otorhinolaryngology – Head and Neck Surgery, University Medical Center Utrecht, PO BOX 85500, 3508 GA Utrecht, The Netherlands; Brain Center Rudolf Magnus, University Medical Center Utrecht, Utrecht, The Netherlands

**Keywords:** Otosclerosis, Stapedotomy, Day-case, Inpatient, Hearing loss, Hearing results, Audiometry, Tinnitus, Vertigo, Quality of life

## Abstract

**Background:**

Otosclerosis is characterized by bony deposits in the otic capsule, resulting in stapes fixation and progressive hearing loss. It can be treated effectively by surgically removing (part of) the stapes and replacing it with a prosthesis. Increasingly, stapes surgery is performed as a day-case procedure. The major drive towards day-case surgery has been out of economic considerations. However, it is also increasingly an explicit patient request and leads to shorter waiting times for surgery, a reduced risk of infection and most likely positively influences the patient’s quality of life as a result of rapid discharge and rehabilitation. Even though stapes surgery seems well suited to a day-case approach, given the low complication rates and early recovery, evidence is scarce and of low quality.

**Methods and design:**

A single-center unblinded randomized controlled trial was designed to (primarily) investigate the effect of hearing outcomes of day-case stapes surgery compared to inpatient stapes surgery and (secondarily) investigate the effect of both methods on quality of life, tinnitus, vertigo and cost-effectiveness. One hundred and twelve adult otosclerosis patients who are eligible for stapes surgery will be randomly assigned to either the day-case or inpatient treatment group. The primary and secondary outcome measures will be assessed using pure-tone audiometry (at approximately 2 months and 1 year follow-up), questionnaires (at 3 months and 1 year follow-up) and costs diaries (weekly the first month after which once a month until 1 year follow-up).

**Discussion/conclusion:**

This trial allows for a comparison between day-case and inpatient stapes surgery to investigate the hypothesis that day-case stapes surgery is associated with a higher quality of life and higher cost-effectiveness, while maintaining equal hearing results, compared to inpatient stapes surgery.

**Trial registration:**

Netherlands Trial Register (www.trialregister.nl): NTR4133, registration date 21^st^ August 2013.

**Electronic supplementary material:**

The online version of this article (doi:10.1186/s12901-016-0024-6) contains supplementary material, which is available to authorized users.

## Background

Otosclerosis is characterized by abnormal sponge-like bone growth in the otic capsule, causing progressive hearing loss, vertigo and/or tinnitus [1]. It mainly affects the ossicular chain and can be treated surgically by removing (part of) the stapes and replacing it with a prosthesis; stapedotomy and stapedectomy respectively. Numerous surgeons have reported either equal hearing results when comparing stapedotomy and stapedectomy or better results with stapedotomy [2–6]. Although stapes surgery has proven to be a safe and effective treatment option for otosclerosis [7], permanent sensorineural hearing loss can occur and is the most dreaded complication of stapes surgery. The incidence of this complication following primary stapes surgery has been reported to be less than 1 % in large series [8, 9]. Other complications associated with stapes surgery are postoperative vertigo in 12-45 % of patients [10–13] and tinnitus in 8-54 % of patients [14–17].

Currently, in our clinic, stapes surgery involves overnight hospital stay. Many other otologic procedures that involved overnight hospital stay in the past are presently being performed on an outpatient basis successfully [18–20]. Ear, nose and throat (ENT) surgery is well suited to a day-case approach as many of the disease entities are benign and procedures are associated with low complication rates [18]. Even though one of the major drives towards day-case surgery has been financial, other non-financial benefits are of major importance. Day-case surgery is associated with shorter waiting time for surgery and reduced risk of infection [21]. Moreover, as a result of a more rapid social and emotional rehabilitation compared to overnight stay, patients might prefer day-case surgery.

Reports on day-case surgery for otosclerosis are scarce [22, 23]. One prospective case series of 24 consecutive otosclerosis patients undergoing day-case stapes surgery [22] reported a readmission rate of 12.5 percent, reasons being vertigo and asthenia. All of these patients were treated under general anesthesia. One prospective case-control study comparing ten day-case and ten inpatient stapedectomies [23] showed no difference in postoperative hearing thresholds, sensorineural hearing loss, speech audiometry or postoperative vertigo. All procedures were performed under local anesthesia. The authors did not report on quality of life (QoL) following day-case surgery.

The lack of (high-quality) studies precludes firm evidence-based recommendations and demonstrates the need for high-quality studies quantifying the benefits of day-case surgery, both clinical and financial. In order to accommodate this need, in this study we shall compare day-case stapes surgery to inpatient stapes surgery. The study will be conducted as a randomized controlled trial.

## Methods and design

This protocol is reported according to the SPIRIT Statement, an international guideline on reporting protocols [24].

### Study objectives

The primary objective of this study is to evaluate effectiveness of day-case stapes surgery compared to in-hospital stapes surgery followed by one-day hospital admittance for otosclerosis, in terms of hearing improvement. In addition, subjective participants’ perception on hearing improvement, QoL, tinnitus, vertigo and cost-effectiveness will be assessed.

### Study design

**Fig. 1:**
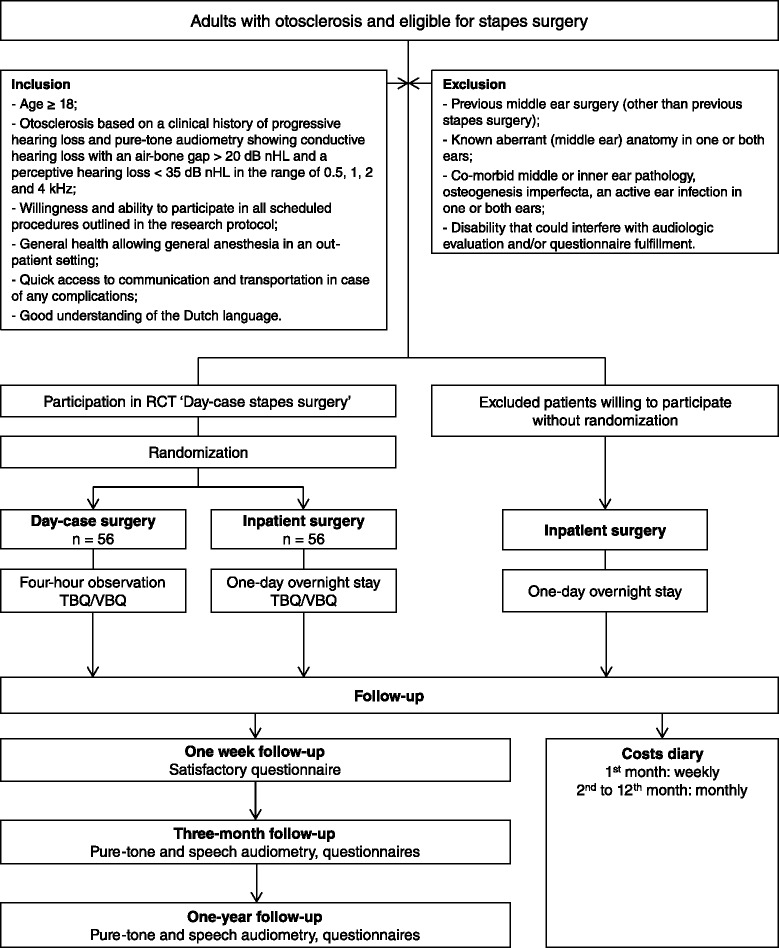
Flow diagram of Day-case stapes surgery study. Abbreviations: RCT = Randomized Controlled Trial, TBQ = Tinnitus Burden Questionnaire, VBQ = Vertigo Burden Questionnaire

The study design will be a single-center, unblinded, randomized controlled trial. Subjects will be assigned to one of two groups: day-case surgery under general anesthesia or inpatient stapes surgery under general anesthesia followed by one-day hospital admittance (Fig. 1).

### Study population

The study population consists of adults with otosclerosis, as diagnosed in the outpatient clinic of the ENT department at the University Medical Center Utrecht (UMC Utrecht), the Netherlands, who will be undergoing (revision) stapedotomy. In order to be eligible to participate in this study, a subject must meet all of the following criteria:

#### Inclusion criteria

- Age ≥ 18.
- Otosclerosis based on a clinical history of progressive hearing loss and pure-tone audiometry showing conductive hearing loss with an air-bone gap > 20 dB above normal adult hearing level and a perceptive hearing loss < 35 dB above normal adult hearing level in the range of 500, 1000, 2000 and 4000 Hz.
- Willingness and ability to participate in all scheduled procedures outlined in the research protocol.
- General health allowing general anesthesia in an outpatient setting as assessed by an anesthesiologist.
- Quick access to communication and transportation in case of any complications.
- Good understanding of the Dutch language.

A potential subject who meets any of the following criteria will be excluded from participation in this study:

#### Exclusion criteria

- Previous middle ear surgery other than previous middle ear inspection or stapes surgery;
- Known aberrant (middle ear) anatomy in one or both ears based on a clinical history, otologic examination, imaging and/or previous middle ear inspection and assessed by an ENT surgeon;
- Co-morbid middle or inner ear pathology based on a clinical history, otologic examination, imaging and/or previous middle ear inspection and assessed by an ENT surgeon, including osteogenesis imperfect or an active ear infection in one or both ears;
- Disability that could interfere with audiologic evaluation and/or questionnaire fulfillment.

### Sample size calculation and recruitment

To establish equivalence in postoperative mean air conduction of 5 dB (standard deviation 10) between the day-case and the inpatient group with an alpha of 0.05 and a power of 80 %, 51 participants per group are needed. To anticipate on a withdrawal of 10 % of the participants, 5 more participants than needed will be recruited per group. At the ENT department in UMC Utrecht, we perform an average of 80 (revision) stapedotomies per year. Assuming a participation rate of 75 %, we will be able to include the necessary number of 112 patients in two years. If participants wish to leave the study or the investigator decides to withdraw a participant from the study for urgent medical reasons, these participants will not be replaced unless these account for more than 10 %.

Patients will be recruited from the outpatient ENT department of the UMC Utrecht. If an otosclerosis patient meets the criteria for stapes surgery and the inclusion criteria for this study, one of the researchers will explain the content of the study and provide the patient with written patient information and an informed consent form. Patients consent to the use of their data for the research purposes outlined in this protocol, which includes publication of the results once the trial has been completed. Further details can be found in Additional file 1 (informed consent form; translated to English, original in Dutch). Patients that do not want to be included in the study because they want to undergo stapes surgery in a clinical setting will be asked whether they want to fulfill the study procedures and whether their data can be used for analysis.

### Randomization, blinding and treatment allocation

A web-based randomization program (Julius Center, UMC Utrecht, Utrecht, The Netherlands) shall be used to allocate subjects randomly into two groups with stratification for age. Block randomization will be used with an allocation ratio of 1:1. The randomization chart, including block size, is established before the start of the study by an independent data manager and will not be available to any of the people involved with enrolment or treatment of participants. Consequently, treatment allocation sequence is concealed for participants, care providers and researchers. Blinding of both participants and care providers is not possible, since both participants and care providers will be aware of the surgical setting and hospital stay.

### Intervention

The surgical procedures, as well as hospitalization in the inpatient group, will take place at the UMC Utrecht. Patients in both groups will undergo (revision) stapedotomy under general anesthesia. The surgical procedure will be performed by three surgeons according to standard protocol in the UMC Utrecht [7].

Patients allocated to the conventional group will be admitted one day before or the day of surgery and will be discharged one day after surgery. Patients allocated to the day-case group will be admitted into the outpatient unit one day before or on the day of the surgery and will be discharged the day of the surgery. Patients are not allowed to drive for 24 h following day-case surgery and will be recommended 24 h of relative bed rest. After a period of 24 h, patients can return to their daily routine. Participants will be asked to contact the hospital in case of severe postoperative vertigo or pain. The ear tampon will be removed in the outpatient clinic seven to ten days postoperatively in both groups.

It is to be expected that patients who have been operated on in day-case will sometimes stay overnight, for example due to postoperative nausea or dizziness. If patients are not physically capable of same-day discharge or if surgeons do not support this, patients will stay overnight. These patients will be asked to complete their follow-up and analyses will be carried out on an intention-to-treat basis.

### Outcome measures

Evaluation will take place preoperatively and at approximately two months, three months and one year postoperatively by means of pure-tone audiometry, speech audiometry and questionnaires. Vertigo and tinnitus will also be evaluated directly postoperatively. In addition participants will be asked to keep a costs diary for the duration of one year. Questionnaires and costs diaries can be fulfilled digitally or on paper and will be sent via email or mail respectively.

#### Primary outcome measure

Our primary outcome is the postoperative air conduction at approximately two and twelve months postoperatively, as measured by pure-tone audiometry for the following frequencies: 500, 1000, 2000 and 4000 Hz, in accordance with the Committee on Hearing and Equilibrium guidelines for the evaluation of results of treatment of conductive hearing loss [25]. However, thresholds at 3000 Hz were substituted in all cases with those at 4000 Hz, because the 3000 Hz frequency is not part of the standard measurement protocol in the Netherlands.

#### Secondary outcome measures

Our secondary outcome measures include (subjective) hearing improvement, patient satisfaction, QoL, tinntus, vertigo and cost-effectiveness.

### Pure-tone and speech audiometry

Pure-tone and speech audiometry will be performed preoperatively and at approximately two and twelve months postoperatively. Measurements will include mean four-frequency bone conduction thresholds, air conduction thresholds and air-bone gap. The following frequencies will be used to calculate mean thresholds: 500, 1000, 2000 and 4000 Hz. Sensorineural hearing loss will be evaluated using frequencies 1000, 2000 and 4000 Hz. Sensorineural hearing loss is defined as a deterioration in mean bone conduction thresholds exceeding 10 to 15 dB.

Performance on speech audiometry will provide information on word recognition abilities and can, in conjunction with pure-tone audiometry, help determine the degree and type of hearing loss.

### Patient satisfaction

Patient satisfaction will be evaluated using the Utrecht patient satisfaction survey (Additional file 2; translated to English, original in Dutch). This seven-item questionnaire was developed in our center and contains questions regarding the hospital stay. Specifically patients are asked whether they were satisfied with the intervention group that they were allocated to.

### Quality of life

QoL and hearing benefit will be assessed preoperatively and at three and twelve months postoperatively using the following four questionnaires:

**-** The Glasgow Health Status Inventory questionnaire: an 18-item questionnaire, which measures the effect of an otologic problem on QoL at the time the questionnaire is completed. Three domains (general, social support and physical health) are measured based on a 5-point Likert scale ranging from high health status to low health status. The total score ranges from 0 to +100.
**-** Glasgow Benefit Inventory: an 18-item questionnaire, which measures the change in health status as a result of a surgical intervention. A specific version of the Glasgow Benefit Inventory will be used that has been validated to measure changes in health status as a result of otorhinolaryngological procedures [26]. The same three domains as the Glasgow Health Status Inventory questionnaire are measured according to the 5-point Likert scale. The total score ranges from -100 (maximal negative benefit), through 0 (no benefit), to +100 (maximum benefit).
**-** EuroQoL-5D: a five-item questionnaire on mobility, self-care, daily activities, pain and complaints and anxiety or depression that assesses general health status [27, 28]. In addition, the general health status is rated on a visual analogue scale than runs from 0 to 10. A score of 0 equals worst imaginable health state and a score of 10 equals best imaginable health state.
**-** Health Utilities Index 3: a fifteen-item questionnaire that measures general health status by evaluating eight domains: vision, hearing, speech, ambulation, dexterity, cognition, emotion and pain [29].

### Tinnitus and vertigo

Tinnitus and vertigo will be assessed preoperatively and at three and twelve months postoperatively using the following four questionnaires. The Utrecht Burden Questionnaire for tinnitus and vertigo will also be administered directly postoperatively in case of direct postoperative tinnitus and/or vertigo:

- Tinnitus Handicap Inventory: a 25-item questionnaire evaluating three domains: a functional, emotional and catastrophic domain [30, 31];
- Tinnitus Questionnaire: a 52-item questionnaire evaluating five domains: tinnitus-related emotional and cognitive distress, intrusiveness, auditory perceptual difficulties, sleep disturbance and somatic complaints. The response categories are ‘true’ (0/2 points), ‘partly true’ (1 point) and ‘not true’ (0/2 points), depending on the question. A validated Dutch version will be used [32, 33];
- Dizziness Handicap Inventory: a 25-item questionnaire evaluating three domains: functional, emotional, and physical aspects of dizziness and unsteadiness. The response categories are ‘yes’ (4 points), ‘sometimes’ (2 points), and ‘no’ (0 points). The total score discriminates between a mild (16–34 points), moderate (36–52 points), and severe (54+ points) handicap. A validated Dutch version will be used [34, 35];
- Utrecht Burden Questionnaire for tinnitus and vertigo: measures severity and character of tinnitus and vertigo by using visual analogue scales and numerical rating scales (Additional file 3).

### Cost-effectiveness/utility analysis

The difference in costs and benefit will be represented using the Incremental Cost Utility/Effectiveness Ratio (ICUR/ICER). The ICUR/ICER is calculated by dividing the difference in costs by the difference in utility or effectiveness. Effectiveness will be reflected by pure-tone audiometric results. Utility reflects the amount of money that people are willing to pay to achieve a certain health status. Utility scores derived from questionnaires such as the EuroQoL-5D and the Health Utilities Index 3 can be used to calculate the ICUR.

Participants will be asked to keep a costs diary. Participants will fulfill this diary preoperatively and at regular intervals postoperatively. The first month the diary will be fulfilled weekly followed by monthly fulfillment for the duration of one year. Costs will be measured from a societal and health care perspective. Both direct and indirect costs will be collected. Direct costs include hospitalization, surgery, doctor’s visits, and diagnostic tests. Indirect costs include travel expenses and sick leave. The Dutch guidelines for costing research in health economic evaluations, issued by the National Healthcare Institute [36], will be used to calculate unit prices of resources that were used.

### Statistical analysis

Baseline characteristics per group will be described as means and standard deviations. Differences in the baseline will be analyzed using the independent samples students *t-*test or non-parametric tests for continuous variables and the Fisher’s exact test for categorical variables.

The primary and secondary outcome data are quantitative and will be presented both continuous and categorical. Between-group mean differences, rate differences and rate ratios with 95 % confidence intervals will be calculated. For further analysis of between-group differences in both primary and secondary outcomes the independent samples students *t–*test or non-parametric tests will be used for continuous outcomes and the Fisher’s exact test for categorical outcomes. Within-subject comparisons will entail differences in mean values and percentages before and after stapes surgery. These will be analyzed using paired *t*-tests for continuous measures and the McNemar test for categorical outcomes.

Missing values will be handled using multiple imputation and all analyses will be performed on an intention-to-treat basis. A sensitivity analysis will be performed using all of the data acquired from patients that opted not to be included in the study, but did fill out the questionnaires and underwent pure-tone audiometric and speech audiometric follow-up.

The data will be reported according to the CONSORT Statement [37, 38].

### Safety reporting, ethics and funding

The study will be conducted according to the principles of the Declaration of Helsinki (Fortaleza, 2013) and in accordance with the Medical Research Involving Human Subjects Act (WMO). The study will be funded by the UMC Utrecht. As such it was peer reviewed by the funding organization. This research protocol was approved by the Institutional Review Board of the UMC Utrecht (NL45219.041.13; version 3, March 2015). The approved research protocol can be found online in the Netherlands Trial Register (www.trialregister.nl): NTR4133, registration date 21^st^ August 2013.

All cases of serious adverse events will be reported to the local Institutional Review Board and adequately followed up. An independent monitor is appointed to check trial quality (completeness of informed consent forms, validity of data, etc.) once a year. All data will be handled confidentially. The data will be analysed anonymously by using a unique patient identification number. The investigator will safeguard the key to the code. The primary source of the data will be paper files, which will be stored in a locked room. The data will be stored on the investigator’s computer as well, which is secured by a password and located in a locked room.

### Trial status

The trial is currently in recruitment phase.

## Discussion/conclusion

Stapes surgery seems to be a surgical procedure that is well suited for day-case treatment as it has proven to be a safe treatment with low complication rates. However, current literature lacks evidence based recommendations supporting day-case stapes surgery. This randomized controlled trial allows for a comparison between day-case and inpatient stapes surgery to investigate the hypothesis that day-case stapes surgery is associated with a higher QoL and higher cost-effectiveness, while maintaining equal hearing results, compared to inpatient stapes surgery. This is the first high quality trial evaluating and quantifying the benefits of day-case stapes surgery for patients with otosclerosis.

## Additional files

Additional file 1:
**Informed consent form.** (PDF 78 kb) Additional file 2:
**Utrecht patient satisfaction survey.** (PDF 23 kb) Additional file 3:
**Utrecht Burden Questionnaire.** (PDF 314 kb)

## Abbreviations

ENT: ear, nose and throat
QoL: quality of life
TBQ: tinnitus burden questionnaire
UMC Utrecht: University Medical Center Utrecht
VBQ: vertigo burden questionnaire
WMO: medical research involving human subjects act
