# Supplementary material for: Day-case stapes surgery: Day-case versus inpatient stapes surgery for otosclerosis: a randomized controlled trial
Source: BMC Ear Nose Throat Disord. 2016 Feb 27;16:3. doi: 10.1186/s12901-016-0024-6 (PMC4769570; doi:10.1186/s12901-016-0024-6)
Supplement: Additional file 1: — Informed consent form. (PDF 78 kb) [file 12901_2016_24_MOESM1_ESM.pdf]

Informed consent form

Day-case versus inpatient stapes surgery for otosclerosis: a randomized controlled trial.

- I have received and read the information brochure (version number 3, 12-03-2015) for participants. I understand the information that is written in the brochure. I had the opportunity to ask additional questions. These questions were answered adequately. I have had plenty of time to consider participation in this study;

- I am aware that participation is completely voluntary. I am aware that I have the possibility to withdraw participation at any moment, without any explanation;

- I am aware that my data are visible for some of the people involved in this study. These people include the researchers, monitors, auditors, etcetera;

- I give permission to use my data for the research purposes as described in the information brochure;

- I am aware that my data will be stored for 15 years following this study and will be destroyed after these 15 years;

- I give the researchers permission to inform my general practitioner about my participation in this study;

- I **will / will not*** give permission to contact me in the future (after this study) and ask me for participation in additional or new research projects;

- I agree to participate in this research project.

Name participant:

Signature: Date: __ / __ / __

I hereby declare that I have fully informed the participant about this research project. I will inform the participant in case of new insight information that could affect the participant’s consent. I will inform the participant in a timely manner.

Name researcher:

Signature: Date: __ / __ / __

---

* Delete as applicable
