# Supplementary material for: Day-case stapes surgery: Day-case versus inpatient stapes surgery for otosclerosis: a randomized controlled trial
Source: BMC Ear Nose Throat Disord. 2016 Feb 27;16:3. doi: 10.1186/s12901-016-0024-6 (PMC4769570; doi:10.1186/s12901-016-0024-6)
Supplement: Additional file 2: — Utrecht patient satisfaction survey. (PDF 23 kb) [file 12901_2016_24_MOESM2_ESM.pdf]

Utrecht patient satisfaction survey

Day-case stapes surgery

Day-case surgery means that you have been admitted one day before or the day of surgery and have been discharged the day of the surgery.

1.	Did you feel more anxious because the surgery was planned in a day-case setting?	Yes	No
2.	Did you feel less anxious because the surgery was planned in a day-case setting?	Yes	No
4.	Did you find it pleasant that you did not have to spend the night in the hospital after the surgery?	Yes	No
3.	If you would have the choice: would you undergo the surgery in day-case setting again next time?	Yes	No
4.	Would you have preferred to have spend the night in the hospital after the surgery?	Yes	No
5.	Would you have preferred to have been admitted the night prior to the surgery?	Yes	No
6.	Were you content with the hospital admittance in general?	Yes	No
7.	How easy or difficult was the first night after the operation on a scale from 0 to 10 (0 is very easy and 10 is as difficult as possible)?<br>0 —————————— 100		

Utrecht patient satisfaction survey

Inpatient stapes surgery
Inpatient surgery means that you have been admitted one day before or the day of surgery followed by one-day hospital admittance.

1.	Did you feel more anxious because the surgery was planned in an inpatient setting?	Yes	No
2.	Did you feel less anxious because the surgery was planned in an inpatient setting?	Yes	No
4.	Did you find it pleasant that you had to spend the night in the hospital after the surgery?	Yes	No
3.	If you would have the choice: would you undergo the surgery in an inpatient setting again next time?	Yes	No
4.	Would you have preferred to have spend the night at home after the surgery?	Yes	No
5.	Would you have preferred to have spend the night prior to the operation at home?	Yes	No
6.	Were you content with the hospital admittance in general?	Yes	No
7.	How easy or difficult was the first night after the operation on a scale from 0 to 10 (0 is very easy and 10 is as difficult as possible)?<br>0 100		
