# Supplementary material for: Day-case stapes surgery: Day-case versus inpatient stapes surgery for otosclerosis: a randomized controlled trial
Source: BMC Ear Nose Throat Disord. 2016 Feb 27;16:3. doi: 10.1186/s12901-016-0024-6 (PMC4769570; doi:10.1186/s12901-016-0024-6)
Supplement: Additional file 3: — Utrecht Burden Questionnaire. (PDF 314 kb) [file 12901_2016_24_MOESM3_ESM.pdf]

Utrecht Burden Questionnaire for tinnitus

First of all

Encircle the number on the thermometer below that summarizes best how much of a burden your tinnitus was in the past week (including today).

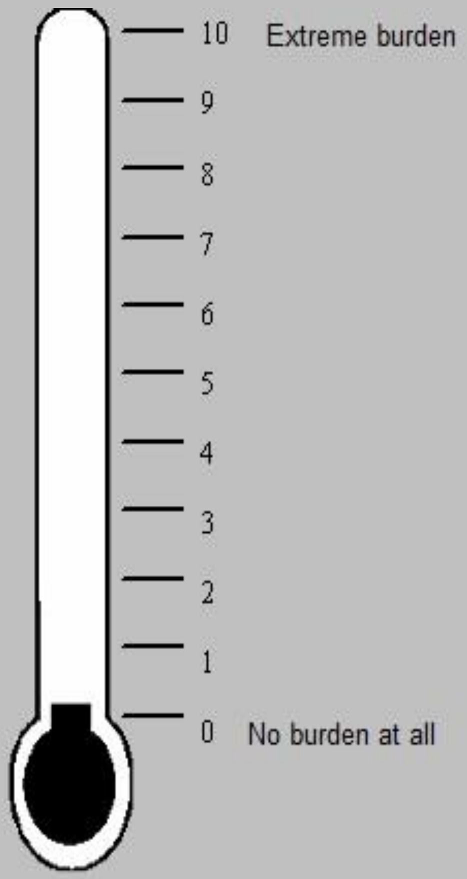

Secondarily

How many sounds does your tinnitus consist of at the moment? ☐ 0 ☐ 1 ☐ 2 ☐ 3 ☐ 4 ☐ 5

Thirdly

Give an indication of how your tinnitus sounds on the scales below. Draw a vertical line through each of the scales. You are allowed to place the vertical line anywhere on the scale. The end of the scale indicates the extreme values. For instance, if you score a loudness of ’10’, this means that the tinnitus cannot be louder. If you hear multiple sounds, you can draw multiple lines on the scale. Please indicate whether the line belongs to the right ear, left ear or within the head, and add numbers if you hear multiple sounds on one side.

Example:

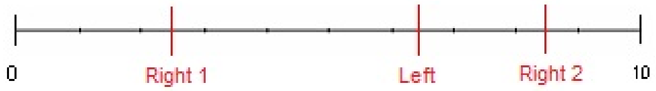

How loud is your tinnitus at this moment?

Inaudible 0 ———————— 10 Extremely loud

How high does your tinnitus sound at this moment?

Extremely low 0 ———————— 10 Extremely high

At what rate was your tinnitus present in the past 24 hours?

Constantly absent 0 ———————— 10 Constantly present

How variable (loudness and/or pitch) has your tinnitus been in the past 24 hours?

Not variable 0 ———————— 10 Extremely variable

Utrecht Burden Questionnaire for tinnitus

Finally

Give an indication on the scales below on whether you have had difficulties or trouble with the following activities in the past week (including today), due to the tinnitus. Draw a vertical line through each of the scales. You are allowed to place the vertical line anywhere on the scale. Take into account that the end of the scale indicates that this could not have been more difficult or given more trouble.

Concentration

No concentration problems — 0 — 100 — Extreme concentration problems

Sleeping

No sleeping problems — 0 — 100 — Extreme sleeping problems

Annoyance

No annoyance — 0 — 100 — Extreme annoyance

Social life

No difficulty in social life — 0 — 100 — Extreme difficulty in social life

Family life

No difficulty in family life — 0 — 100 — Extreme difficulty in family life

Work / study

No difficulty with work / study — 0 — 100 — Extreme difficulty with work / study

O Not applicable

Utrecht Burden Questionnaire for vertigo

First of all

Encircle the number on the thermometer below that summarizes best how much of a burden your vertigo was in the past week (including today).

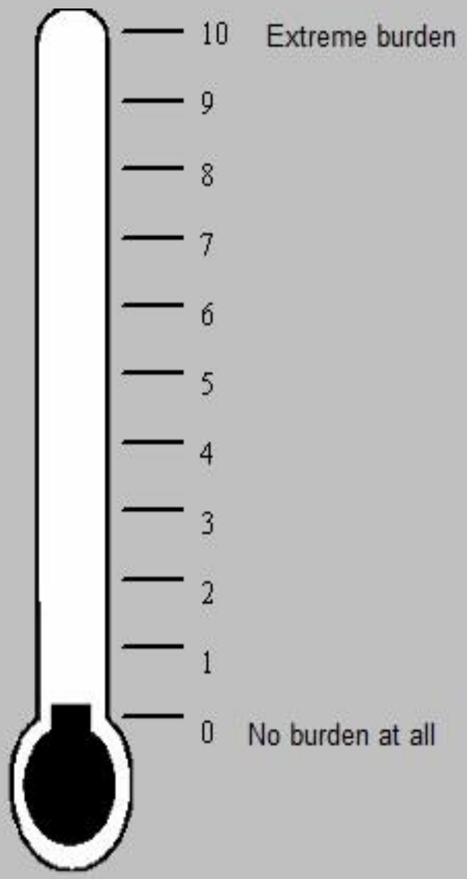

Secondarily

Answer the questions below about how the dizziness is best described (multiple answers are possible).

- **How severe is your dizziness at this moment?**

Absent 0 —————————————— 100 Extremely severe

- **Type of dizziness?**

☐ Vertigo ☐ Lightheadedness ☐ Feeling of fainting

☐ Other:..

- **Provoking factors?**

☐ Head movements ☐ Change of body position ☐ Loud noises

☐ When standing up ☐ During excercise ☐ Unknown

☐ Other:..

- **Additional complaints?**

☐ Nausea ☐ Vomiting ☐ Hearing complaints ☐ Tinnitus

☐ None ☐ Other:...

- **Aspect of the dizziness?**

☐ Constantly present ☐ Attack → give an indication of the duration:.........................

☐ Other:..

Utrecht Burden Questionnaire for vertigo

Finally

Give an indication on the scales below on whether you have had difficulties or trouble with the following activities in the past week (including today), due to the tinnitus. Draw a vertical line through each of the scales. You are allowed to place the vertical line anywhere on the scale. Take into account that the end of the scale indicates that this could not have been more difficult or given more trouble.

Concentration

No concentration problems — 0 ———————— 100 — Extreme concentration problems

Sleeping

No sleeping problems — 0 ———————— 100 — Extreme sleeping problems

Annoyance

No annoyance — 0 ———————— 100 — Extreme annoyance

Social life

No difficulty in social life — 0 ———————— 100 — Extreme difficulty in social life

Family life

No difficulty in family life — 0 ———————— 100 — Extreme difficulty in family life

Work / study

No difficulty with work / study — 0 ———————— 100 — Extreme difficulty with work / study

O Not applicable
